# Impact of Decitabine Conditioning on Allo‐HSCT Outcomes in AML and Intermediate‐to‐High‐Risk MDS Patients in Remission

**DOI:** 10.1002/cam4.71081

**Published:** 2025-07-23

**Authors:** Shuling Yu, Wanchuan Zhuang, Shengfa Gao, Tongyu Li, Xiao Yan, Guifang Ouyang, Ping Zhang

**Affiliations:** ^1^ Department of Hematology The First Affiliated Hospital of Ningbo University Ningbo China; ^2^ Health Science Center, Ningbo University Ningbo China; ^3^ Department of Hematology Lianyungang Second People's Hospital Lianyungang Jiangsu China

**Keywords:** allogenic hematopoietic stem cell transplantation, AML, decitabine, graft‐versus‐host disease, MDS

## Abstract

**Background:**

Allogeneic hematopoietic stem cell transplantation (allo‐HSCT) remains the sole curative option for myeloid malignancies, though high toxicity from conditioning regimens and complications like graft‐versus‐host disease (GVHD) limit its success. The potential benefit of incorporating decitabine (DAC) into conditioning regimens for acute myeloid leukemia (AML) and intermediate‐to‐high‐risk myelodysplastic syndromes (MDS) patients in remission remains unclear.

**Methods:**

We conducted a retrospective, single‐center study analyzing data from January 2016 to December 2020 at the First Affiliated Hospital of Ningbo University with a median follow‐up of 45.05 months (range, 1–96 months). Outcomes were compared between patients receiving DAC+HSCT versus HSCT alone, with primary endpoints of 5‐year overall survival (OS), progression‐free survival (PFS), and relapse rate. Secondary analyses examined outcomes by remission status (CR1 vs. others) and age subgroups (< 31.5 years). Immune cell subsets (CD3−CD56+ NK cells) were evaluated for GVHD correlation.

**Results:**

The DAC+HSCT group exhibited 5‐year OS of 51.9% and PFS of 46.1%, compared to 67% OS and 56.5% PFS in the HSCT‐only group. The 5‐year relapse rate was 16.9% for DAC+HSCT versus 23.2% for HSCT alone. DAC did not significantly improve outcomes in complete remission (CR1) patients but improved OS and PFS in patients under 31.5 years of age. Elevated CD3−CD56+ NK cells in the DAC+HSCT group were associated with higher incidence of severe acute GVHD (aGVHD).

**Conclusion:**

While DAC conditioning did not provide overall survival benefit for AML/MDS patients undergoing allo‐HSCT, it improved outcomes in younger individuals (< 31.5 years). Higher NK cell proportions may serve as a potential biomarker for early aGVHD intervention, warranting further investigation into risk‐stratified conditioning approaches.

**Abbreviations**
aGVHD: acute GVHD
allo‐HSCT: allogeneic hematopoietic stem cell transplantation
AML: acute myeloid leukemia
AML‐MRC: AML with myelodysplasia‐related changes
Aza: azacitidine
cGVHD: chronic GVHD
CIR: cumulative incidence of relapse
CIs: confidence intervals
CMV: cytomegalovirus
CR: complete remission
CRR: cumulative relapse rate
CsA: cyclosporin
DAC: decitabine
EBV: Epstein–Barr virus
ECOG PS: Eastern Cooperative Oncology Group performance status
GRFS: GVHD‐free relapse‐free survival
GVHD: graft‐versus‐host disease
GVL: graft‐versus‐leukemia
HCT‐CI: Hematopoietic Cell Transplantation Comorbidity Index
HMAs: hypomethylating agents
HRs: hazard ratios
KM: Kaplan–Meier
MAC: myeloablative conditioning
MDS: myelodysplastic syndromes
MMF: mycophenolate mofetil
MPN: myeloproliferative neoplasms
MTX: methotrexate
NR: non‐response
NRM: non‐relapse mortality
OS: overall survival
PFS: progression‐free‐survival
RFS: relapse‐free survival
RIC: reduced‐intensity conditioning
SHRs: subdistribution hazard ratios
STRs: short tandem repeats
TRM: transplant‐related mortality

## 1 Introduction

Myelodysplastic syndromes (MDS) and acute myeloid leukemia (AML) are hematologic malignancies for which allogeneic hematopoietic stem cell transplantation (allo‐HSCT) remains the only curative treatment, offering long‐term survival to over half of patients [1, 2]. However, despite its potential, the high incidence of complications, including disease relapse, graft‐versus‐host disease (GVHD), and infections, limits its widespread use. To address these challenges, many stem cell transplantation centers have focused on optimizing conditioning regimens [2].

To date, the FDA has approved two hypomethylating agents (HMAs), decitabine (DAC) and azacitidine (Aza), for MDS treatment. DAC integrates into replicating DNA, irreversibly inhibiting DNA methyltransferase I, which leads to leukemic cell differentiation and the epigenetic silencing of potential tumor antigens [3]. DAC has demonstrated efficacy in initial induction chemotherapy for elderly patients with AML exhibiting myelodysplasia‐related changes (AML‐MRC)/MDS, in maintenance therapy for these patients, and in reinduction for relapsed cases [4, 5, 6]. The addition of demethylating agents to standard chemotherapy regimens may improve outcomes, even in the absence of methylated gene mutations [7]. Previous research demonstrated that DAC as part of a pretreatment regimen for high‐risk patients with MDS resulted in a lower 3‐year cumulative incidence of relapse (CIR) and improved overall survival (OS) [8].

Moreover, DAC's role in allo‐HSCT extends beyond myelosuppression. A phase II open‐label study evaluating D‐CAG induction therapy for elderly patients with newly diagnosed AML found that DAC, in combination with other chemotherapeutic agents, effectively eradicated leukemic cells [9]. DAC has also been shown to enhance the graft‐versus‐leukemia (GVL) effect and prevent GVHD following allo‐HSCT [2]. HMAs have been proven effective in induction and consolidation regimens prior to allo‐HSCT, leading to low relapse rates, mild GVHD, and favorable survival outcomes [10, 11]. Incorporating DAC into treatment regimens for patients with MDS and MDS/myeloproliferative neoplasms (MPN) results in tolerable toxicity [12].

Despite these advances, there is no empirical evidence comparing the effectiveness of DAC‐containing conditioning regimens in allo‐HSCT for intermediate‐to‐high‐risk patients with MDS and those with AML in complete remission (CR) 1 stage. Additionally, long‐term real‐world studies (over 3 years) comparing DAC‐containing regimens with non‐DAC regimens in these myeloid malignancies are lacking [13]. This study aims to investigate the long‐term survival outcomes, including OS and progression‐free survival (PFS), associated with the addition of DAC to HSCT conditioning regimens in AML and intermediate‐to‐high‐risk patients with MDS.

## 2 Materials and Methods

This study involved 76 patients with myeloid neoplasms, including AML and high‐intermediate risk MDS, who underwent allo‐HSCT at The First Affiliated Hospital of Ningbo University between January 2016 and December 2020. The study was approved by the Institutional Review Board of the First Affiliated Hospital of Ningbo University (2024‐207RS) and was conducted in accordance with the principles outlined in the Declaration of Helsinki. Given the retrospective nature of the study, the medical ethics committee of the First Affiliated Hospital of Ningbo University waived the requirement for written informed consent.

### 2.1 Inclusion Criteria

There were no specific restrictions regarding age or gender. All patients were required to have completed pre‐transplant evaluations, including routine blood tests, bone marrow cytology, flow cytometry, chromosomal karyotype analysis, and other relevant assessments. Diagnoses had to align with the World Health Organization classification criteria for myeloid tumors and acute leukemia. Additionally, a comprehensive pre‐transplant evaluation was mandatory.

### 2.2 Exclusion Criteria

Patients who died prior to HSCT were excluded. Also excluded were individuals with hematologic disorders other than AML or high‐risk MDS, as well as those with severe vital organ dysfunction that rendered them ineligible for allo‐HSCT.

Various conditioning regimens were tailored to the individual patient's circumstances. These included myeloablative conditioning (MAC) with the BU/CY regimen (busulfan 3.2 mg/m^2^ from days −7 to −4 and cyclophosphamide 60 mg/kg from days −3 to −2), reduced‐intensity conditioning (RIC)‐Flu‐BUCY‐AraC regimen (fludarabine 30 mg/m^2^ from days −9 to −5, busulfan 3.2 mg/m^2^ from days −9 to −8, cyclophosphamide 30 mg/kg from days −5 to −4, and cytarabine 2 g/m^2^ from days −3 to −2), and the RIC‐BUCY‐AraC regimen (busulfan 3.2 mg/m^2^ from days −8 to −6, cyclophosphamide 60 mg/kg from days −5 to −4, and cytarabine 2 g/m^2^ from days −3 to −2). DAC (15 mg/m^2^) was administered during the first 3 days of the conditioning regimen according to a specialized protocol. This dosage and duration were selected to balance epigenetic modulation effects while minimizing the risks of myelosuppression, based on previous studies showing that low‐dose DAC could maintain hypomethylating activity while reducing toxicity.

Immunosuppressants including cyclosporine (CsA), mycophenolate mofetil (MMF), and methotrexate (MTX) were utilized to prevent GVHD.

### 2.3 Key Endpoints in Clinical Outcomes

OS, defined as the time from randomization to death from any cause, is considered the primary efficacy endpoint in clinical trials, especially when survival is sufficiently prolonged.

PFS, defined as the time from randomization to disease progression or death from any cause, includes progression as a key event. While PFS often precedes death, it is typically shorter than OS and can be assessed earlier, reducing the follow‐up period.

Non‐relapse mortality (NRM) is defined as mortality unrelated to disease recurrence or progression during the observation period.

Cumulative relapse rate (CRR) represents the probability that a patient will experience a recurrence of the primary disease within a specific time frame following hematopoietic stem cell transplantation.

CIR, defined as the probability of relapse over time, accounting for relapse and competing risks like non‐relapse mortality.

### 2.4 Statistics

The enrollment cutoff was December 31, 2020, with the final follow‐up on December 31, 2023. The median follow‐up duration was 45.05 months (range: 1–96 months). Continuous quantitative data with normal distribution were expressed as mean ± standard deviation, and group comparisons were performed using the *t*‐test. For non‐normally distributed data, results were presented as median [P25, P75], with group comparisons conducted using the rank‐sum test. Categorical data were described as frequencies (percentages), and group comparisons were made using the chi‐square test or Fisher's exact test. Logistic regression models were employed to estimate odds ratios and 95% confidence intervals (CIs) for binary outcomes, adjusting for age, sex, Eastern Cooperative Oncology Group (ECOG) score, and diagnosis in the treatment group model, and for age, sex, ECOG score, and treatment factors in the diagnosis group model. For repeated‐measure outcomes, such as platelet count, differences between groups were estimated using the generalized estimating equation model from the R package “geepack,” employing an “exchangeable” correlation structure. The model included time, group, and time × group interaction terms, adjusted for covariates and baseline values. Line plots were generated to visualize the estimated means or relative changes from baseline at different time points for each group. All statistical analyses and graphical representations were performed using R (version 4.4.1). A two‐sided *p*‐value of < 0.05 was considered statistically significant.

## 3 Results

### 3.1 Clinical Characteristics of Participants

**FIGURE 1:**
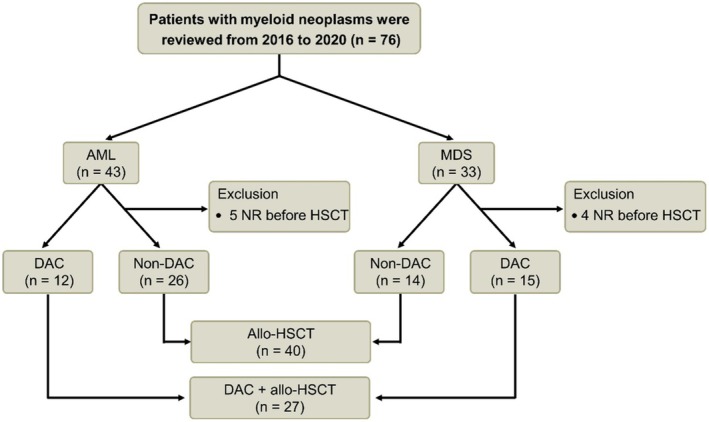
Flow diagram of enrolled patients. A total of 76 patients with myeloid neoplasms were included in the study, consisting of 43 patients with AML and 33 patients with MDS.

A total of 76 patients with myeloid neoplasms, including AML and high‐intermediate risk MDS, who underwent allo‐HSCT at our institution between January 2016 and December 2020, were enrolled. Nine patients were excluded due to non‐response (NR) status before HSCT. Ultimately, 27 patients who received DAC as part of their allo‐HSCT pretreatment regimen were included, comprising 12 patients with AML and 15 patients with MDS. The HSCT group included 26 patients with AML and 14 patients with MDS. The patient selection process is illustrated in Figure 1. Statistical analysis revealed no significant differences in sex ratio or age distribution between the two groups, as shown in Table 1.

**TABLE 1:** Patient demographics and clinical characteristics.

Characteristics	DAC + HSCT (*n* = 27)	HSCT (*n* = 40)	*p*
Age median (range)	42.39 ± 2.4	43.78 ± 2.69	0.618
Sex			0.625
Male (%)	16 (59.3%)	21 (52.5%)	
Female (%)	11 (40.7%)	19 (47.3%)	
Histology			0.123
AML (%)	12 (44.4%)	26 (63.4%)	
MDS (%)	15 (55.6%)	14 (36.6%)	
DRI			0.307
Low risk	2 (7.4%)	8 (20%)	
Intermediate risk	17 (63.0%)	24 (60%)	
High risk	8 (29.6%)	8 (20%)	
AML			0.640
Favorible	2 (16.7%)	8 (30.8%)	
Intermediate	8 (66.7%)	15 (57.7%)	
Adverse	2 (16.7%)	3 (11.5%)	
IPSS‐R classification (%)			0.812
Intermediate	9 (60%)	9 (64.3%)	
High risk	6 (40%)	5 (35.7%)	
ECOG PS at baseline			0.458
0–1 (%)	18 (66.7%)	30 (75%)	
2 (%)	9 (33.3%)	10 (25%)	
HCT‐CI			1
0 (%)	23 (85.2%)	34 (85%)	
1 (%)	4 (14.8%)	5 (12.5%)	
2 (%)	0 (0%)	1 (2.5%)	
Male recipient and female donor			0.609
Yes (%)	7 (25.9%)	12 (31.7%)	
No (%)	20 (74.1%)	28 (68.3%)	
Donor type			0.924
Unrelated donor (%)	5 (18.5%)	6 (15%)	
Haploidentical donor (%)	12 (44.4%)	18 (45%)	
Matched sibling (%)	10 (37%)	16 (40%)	
Graft source			0.655
PB (%)	15 (55.6%)	20 (50%)	
BM + PB (%)	12 (44.4%)	20 (50%)	
Conditioning regimen			0.533
Myeloablative (MA)	26 (97%)	38 (94.4%)	
Reduced intensity (RIC)	1 (3%)	2 (5.6%)	
Grafts			
MNC (10^6^/kg)	7.49 ± 3.04	6.77 ± 1.74	0.222
CD34 (10^6^/kg)	3.31 ± 2.12	4.08 ± 4.04	0.333

### 3.2 The Survival Profile of the DAC‐HSCT Group Versus the HSCT Group

**FIGURE 2:**
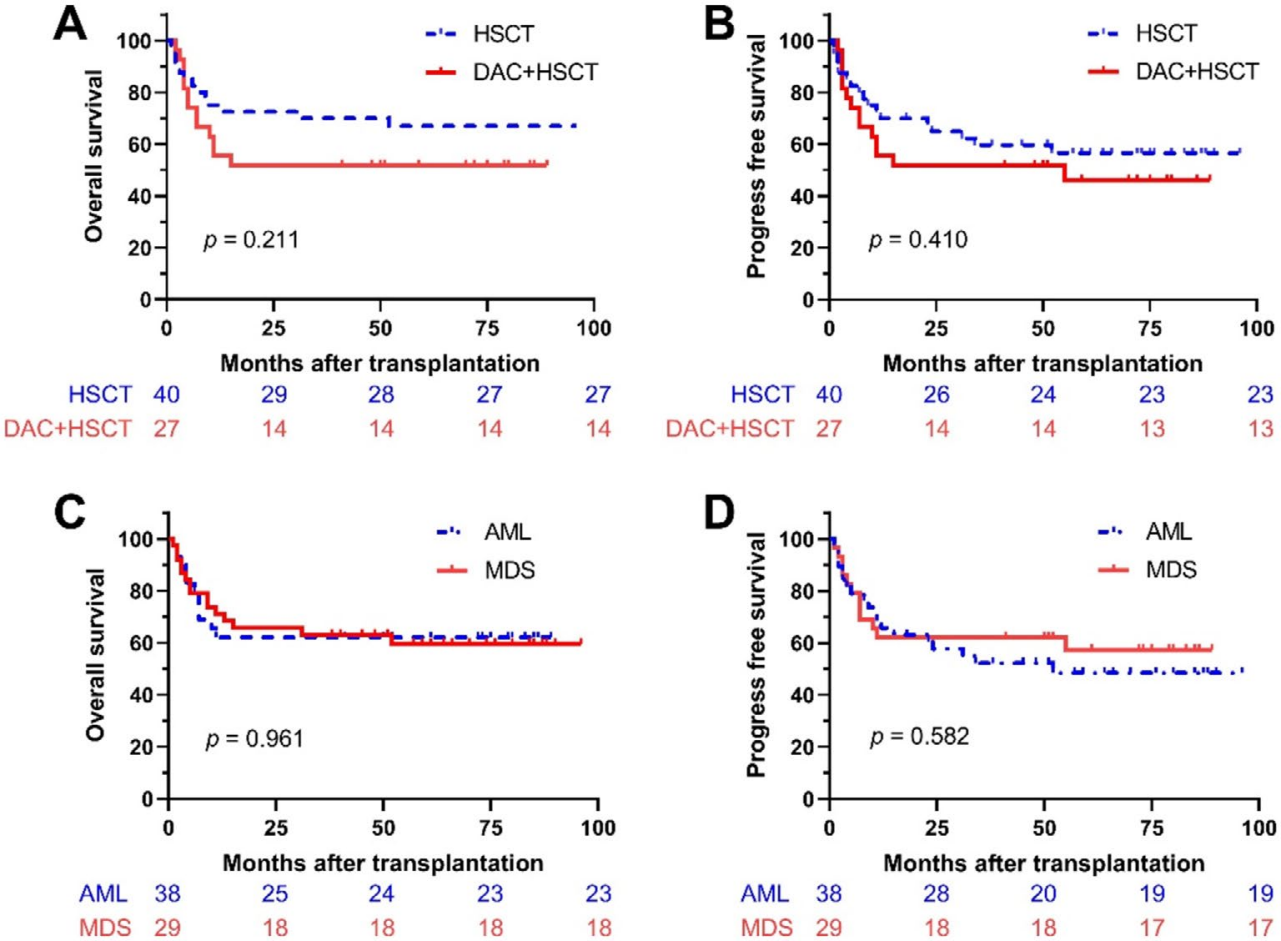
Survival outcomes in different groups. Kaplan–Meier survival curves for OS (A) and PFS (B) in the DAC + HSCT and HSCT groups; OS (C) and PFS (D) in patients with AML and MDS.

Among 67 patients, the 5‐year OS rate was 60.7%, with a median OS of 61.9 ± 5.27 months (95% CI, 51.57–71.24 months). The 5‐year PFS rate was 52.5%, with a median PFS of 56.5 ± 5.27 months (95% CI, 46.18–66.83 months). In the DAC + HSCT group, the 5‐year OS was 51.9% (95% CI: 36.1–74.6), while in the HSCT group, it was 67.0% (95% CI: 53.7–83.5). The 5‐year PFS in the DAC + HSCT group was 46.1% (95% CI: 30.0–70.9), compared to 56.5% in the HSCT group (95% CI: 42.8–74.6). No significant differences in OS or PFS were observed between the two groups (Figure 2A,B).

When comparing AML and MDS cohorts, the 5‐year OS for patients with AML was 60.5% (median OS: 62.04 ± 6.9 months, 95% CI: 48.51–75.56), while for patients with MDS, it was 62.1% (median OS: 57.37 ± 7.52 months, 95% CI: 42.62–72.11). The 5‐year PFS for patients with AML was 50.0% (median PFS: 53.87 ± 6.92 months, 95% CI: 40.3–67.43), compared to 58.6% for patients with MDS (median PFS: 55.72 ± 7.46 months, 95% CI: 41.11–70.34). No significant differences in OS or PFS were observed between the two groups (Figure 2C,D). The 5‐year cumulative relapse rate for patients with AML was 24.8% (95% CI: 11.9–40.1), while for patients with MDS, it was 15.1% (95% CI: 4.4–31.8). NRM was 17.3% for patients with AML (95% CI: 6.7–32.1) and 22% for patients with MDS (95% CI: 8.5–39.5). The 5‐year GVHD‐free relapse‐free survival (GRFS) was 13.9% for patients with AML (95% CI: 4.2–45.9) and 39.4% for patients with MDS (95% CI: 22.8–68.1).

**FIGURE 3:**
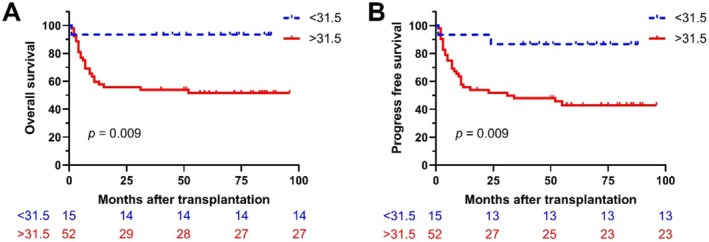
Survival outcomes by age group (above and below 31.5 years). Kaplan–Meier survival curves for OS (A) and PFS (B).

Further analysis using receiver operating characteristic (ROC) curves determined the optimal cutoff age at 31.5 years. Kaplan–Meier analysis demonstrated superior OS and PFS in younger patients (< 31.5 years) compared to older patients (≥ 31.5 years) (Figure S1). Among patients under 31.5 years, those receiving DAC + HSCT had better OS than those treated with HSCT alone (Figure 3). Conversely, in patients older than 31.5 years, DAC + HSCT resulted in poorer OS than HSCT alone.

### 3.3 Graft‐Versus‐Host Disease After Transplantation

The overall incidence of acute GVHD (aGVHD) post‐transplant was 43.28% (29/67). Among these, 24 patients had Grade I–II aGVHD, and 5 had Grade III–IV. In the DAC + HSCT cohort, 9 patients (33.3%, 9/27) developed aGVHD, with 29.6% (8/27) having Grade I–II and 3.7% (1/27) having Grade III–IV (*p* > 0.05). In the HSCT group, 20 patients experienced aGVHD, with 40% (16/40) of cases being Grade I–II and 10% (4/40) Grade III–IV (*p* > 0.05). Chronic GVHD (cGVHD) occurred in 14.8% (4/27) of the DAC + HSCT group and 25% (10/40) of the HSCT group (*p* = 0.373, Table S1).

**FIGURE 4:**
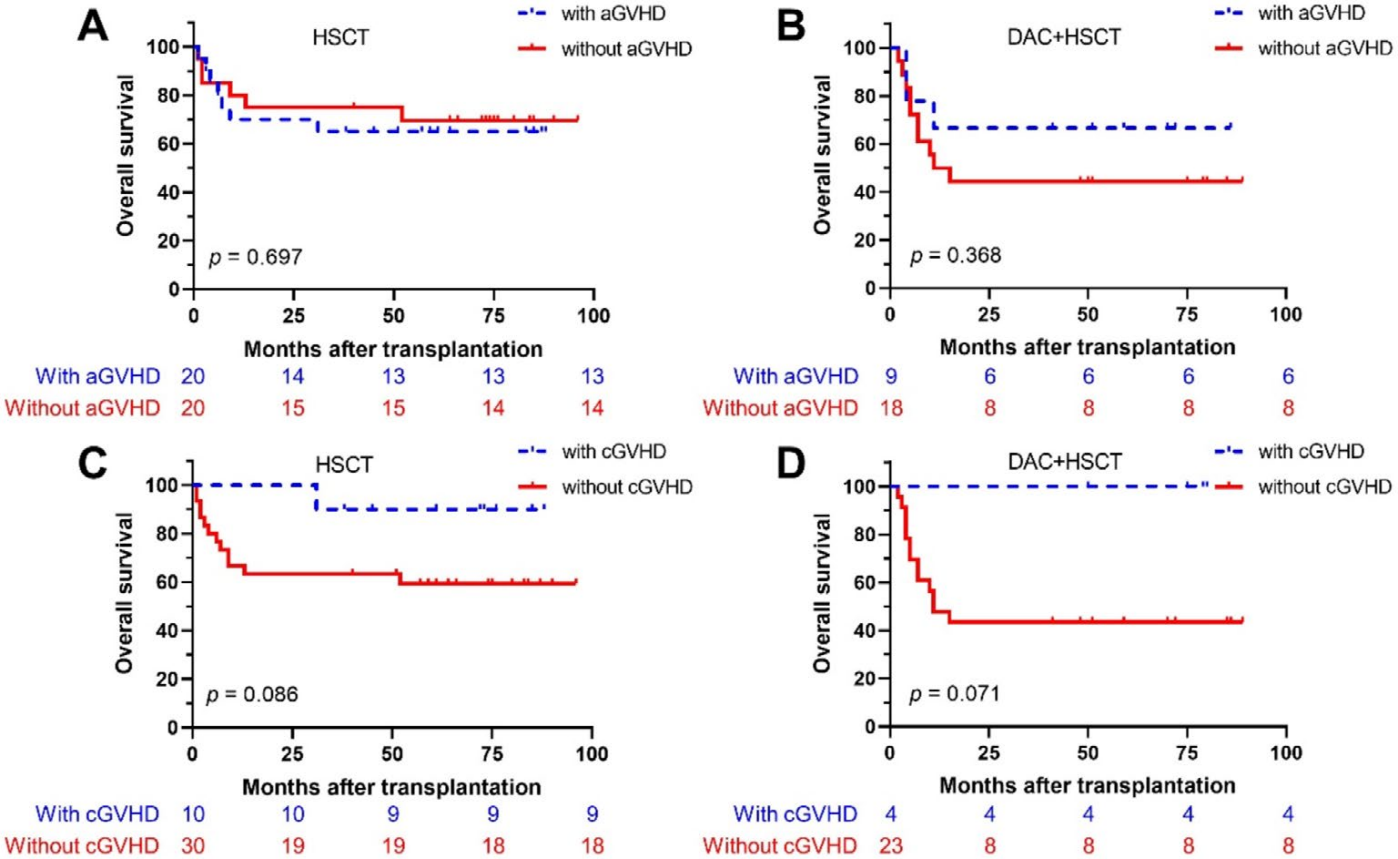
Survival outcomes for patients with or without aGVHD and cGVHD. (A, B) aGVHD in the HSCT and DAC + HSCT groups, respectively; (C, D) cGVHD in the HSCT and DAC + HSCT groups.

Survival outcomes were compared between patients with and without aGVHD in both groups. In the HSCT cohort, the 5‐year OS for patients with aGVHD was 65% (13/20), with a median OS of 60.21 ± 8.56 months (95% CI: 43.42–76.99). In contrast, patients without aGVHD had a 5‐year OS of 70% (14/20), with a median OS of 70.99 ± 8.78 months (95% CI: 53.77–88.21). In the DAC + HSCT group, the 5‐year OS for patients with aGVHD was 66.7% (6/9), with a median OS of 59.33 ± 12.59 months (95% CI: 34.66–84.01). For patients without aGVHD, the 5‐year OS was 44.4% (10/18), with a median OS of 43.36 ± 9.65 months (95% CI: 24.46–62.27) (Figure 4A,B). Survival analysis for patients with aGVHD revealed superior outcomes, but differences were not statistically significant (Figure 4C,D). The 5‐year OS for patients with aGVHD was 100% (4/4) in the DAC + HSCT group, while in the DAC + HSCT cohort, the 5‐year OS for patients with aGVHD was 90% (9/10).

### 3.4 Relapse and Other Adverse Effects After Allo‐HSCT

The 5‐year CRR was 16.9% (95% CI: 4.8–35.3) in the DAC + HSCT group, compared to 23.2% (95% CI: 11.2–37.6) in the HSCT group. The 5‐year NRM rate was 21.3% (95% CI: 6.9–40.9) in the DAC + HSCT group, and 18.3% (95% CI: 7.9–32.2) in the HSCT group. No significant differences in CRR or NRM were observed between the two groups. The 5‐year GRFS was not available for the DAC + HSCT group but was 26.7% (95% CI: 11.9–59.8) in the HSCT group.

The most prevalent side effects, apart from GVHD, were infections and neutropenia resulting from myelosuppression. Grade III–IV hematological adverse effects occurred in 65 patients. The average recovery times for agranulocytosis and thrombocytopenia were 15 and 20.5 days, respectively, in the DAC + HSCT group, and 15 and 19 days, respectively, in the HSCT group. Forty‐three patients (62.3%) developed infections, with 21 (63.6%) in the DAC + HSCT group and 22 (61.1%) in the HSCT group. The most common infection sites included pulmonary (24/43, 46.5%), urinary tract (10/43, 23.3%), gastrointestinal tract (5/43, 11.6%), and skin (5/43, 11.6%). Cytomegalovirus (CMV) infection occurred in 10 patients (23.3%). Among infected patients, 17 had gram‐positive infections (39.5%), 19 had gram‐negative infections (44.2%), and 11 had fungal infections (25.6%). No treatment‐related deaths were reported.

The rates of CMV infection were 75.8% in the DAC + HSCT group and 80.6% in the HSCT group, with no significant difference. The Epstein–Barr virus (EBV) positivity rate was 30.3% for DAC + HSCT patients and 50% for HSCT patients, with all viral loads below 1 × 10^6^/L. No statistically significant difference was found between the groups (Figure S2A,B). A one‐way ANOVA for OS and PFS, incorporating variables such as gender, age, disease diagnosis, ECOG score, Hematopoietic Cell Transplantation Comorbidity Index (HCT‐CI) score, ATG use, donor source, and stem cell source, is presented in Table 2. Multifactorial analysis identified age and ECOG score as the primary determinants of OS and PFS (Table 3).

**TABLE 2:** One‐way analysis of variance for OS and PFS.

Characters	OS		PFS	
	HR (95% CI)	*p*	HR (95% CI)	*p*
Sex (female vs. male)	1.284 (0.361–1.68)	0.524	1.38 (0.682–2.791)	0.371
Age (< 31.5 vs. > 31.5)	0.403 (0.169–0.96)	0.04	0.428 (0.196–0.931)	0.002
Other combinations vs. female to male	0.837 (0.352–1.993)	0.688	0.766 (0.342–1.715)	0.517
Diagnosis (AML vs. MDS)	1.017 (0.467–2.216)	0.965	1.226 (0.595–2.528)	0.58
ECOG 0–1 vs. > 2	0.32 (0.147–0.694)	0.004	0.336 (0.163–0.693)	0.003
HCT‐CI 0 vs. > 1	1.427 (0.428–4.752)	0.563	1.235 (0.432–3.531)	0.693
ATG used vs. unused	0.850 (0.394–1.834)	0.679	0.798 (0.394–1.614)	0.530
Donor				
MRD		0.348		0.23
HID	0.817 (0.366–1.82)	0.620	3.661 (0.832–116.135)	0.086
MUD	0.33 (0.074–1.479)	0.148	3.138 (0.717–13.726)	0.129
Stem cell source (PB vs. PB + BM)	0.917 (0.425–1.979)	0.825	0.741 (0.365–1.504)	0.407
DAC + HSCT vs. HSCT	1.623 (0.751–3.51)	0.22	1.345 (0.662–2.732)	0.415

**TABLE 3:** Multifactorial analysis of OS and PFS.

Characters	OS		PFS	
	HR (95% CI)	*p*	HR (95% CI)	*p*
DAC + HSCT vs. HSCT	0.717 (0.33–1.556)	0.4	0.87 (0.426–1.776)	0.701
Age	7.927 (1.069–58.809)	0.043	5.262 (1246–22.217)	0.024
ECOG	2.939 (1.351–6.395)	0.007	2.917 (1.409–6.04)	0.004

### 3.5 Reconstitution of the Immune System Across Different Groups

Median times to neutrophil and platelet engraftment were comparable between the DAC + HSCT and HSCT groups (neutrophil engraftment: 14.7 ± 2.64 days vs. 14.85 ± 4.32 days; platelet engraftment: 20.63 ± 2.74 days vs. 18.54 ± 1.82 days). Semiquantitative fluorescent PCR analysis of short tandem repeats (STRs) confirmed complete chimerism in all patients, with chimerism levels exceeding 95% by day 28.

Flow cytometry analysis at Day +28 revealed no significant differences between the DAC + HSCT and HSCT groups in terms of CD4+ T cells, CD8+ T cells, CD4/CD8 ratio, CD4+CD25+ Treg cells, and CD3−CD56+ NK cells (Figure S3). Moreover, no significant differences were observed in the proportions of CD4+CD25+ Treg cells or CD3−CD56+ NK cells between long‐term survivors in either group (Figure S4), suggesting that NK and Treg cell proportions did not influence long‐term survival, irrespective of the preconditioning regimen.

**FIGURE 5:**
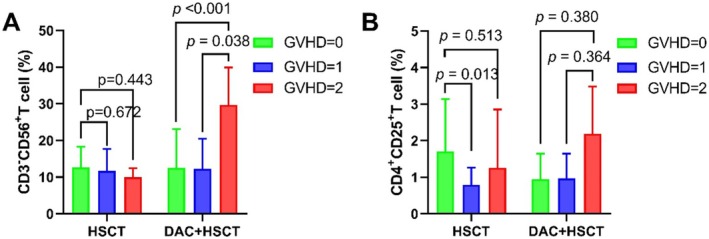
Percentages of CD3−CD56+ NK cells (A) and CD4+CD25+ Treg cells (B) in the DAC + HSCT group versus the HSCT group in relation to different degrees of aGVHD.

In the DAC + HSCT group, NK cell percentages were significantly higher in patients with Grade III–IV aGVHD compared to those without GVHD or with Grade I–II aGVHD. A similar trend was observed for Treg cells, though not reaching statistical significance (Figure 5).

### 3.6 Comparison of Biochemical Markers Between Groups Post‐Transplantation

The impact of the DAC‐containing conditioning regimen on liver and kidney function, along with other biochemical markers, was also assessed post‐transplantation. No significant differences were observed in the trends of ALT, AST, GGT, ADA, creatinine, and LDH levels between the two groups from pre‐transplant to 28 days post‐transplant. A detailed comparison is provided in Figure S5.

### 3.7 Competing Risks Analysis of Survival Outcomes

**FIGURE 6:**
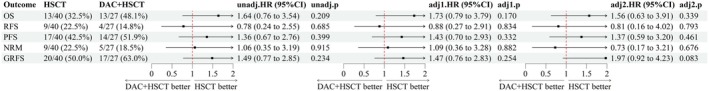
Multivariate COX analysis of survival outcomes. GRFS, GVHD relapse‐free survival; NRM, non‐relapse mortality; OS, overall survival; PFS, progression‐free survival; RFS, cumulative relapse rate.

Survival outcomes were assessed using Kaplan–Meier (KM) curves, with inter‐group comparisons conducted via the log‐rank test. Cumulative incidence at various time points was calculated, and hazard ratios (HRs) with 95% CIs were estimated through Cox regression models. Three models were employed: Model 1 (no adjustment), Model 2 (adjusted for age and gender), and Model 3 (further adjusted for ECOG performance status, transplant type, and female donors paired with male recipients). Multivariate Cox regression analysis revealed no statistically significant difference in OS between the groups (HR = 1.56, 95% CI: 0.63–3.91, *p* = 0.339), after adjusting for diagnostic variables. Similarly, no significant differences were observed for relapse‐free survival (RFS) (HR = 0.81, 95% CI: 0.16–4.02, *p* = 0.793), PFS (HR = 1.37, 95% CI: 0.59–3.20, *p* = 0.461), NRM (HR = 0.73, 95% CI: 0.17–3.21, *p* = 0.676), and GRFS (HR = 1.97, 95% CI: 0.92–4.23, *p* = 0.083) (Figure 6).

A competing risks analysis for RFS, NRM, and GRFS was also performed. Cumulative incidence curves were plotted, and differences between groups were compared using the Fine‐Gray method. Competing risks Cox regression was employed to estimate subdistribution HRs (SHRs) and 95% CIs, adjusting for the same variables as in the Cox models. The results showed no significant differences in RFS, NRM, and PFS between the groups (SHRs: 0.44, 95% CI: 0.07–2.72, *p* = 0.376; 0.48, 95% CI: 0.10–2.36, *p* = 0.367; 1.64, 95% CI: 0.71–3.77, *p* = 0.245) (Figure S6).

## 4 Discussion

Epigenetics has emerged as a pivotal research domain, with therapies including HMAs, histone deacetylase inhibitors, immunomodulatory drugs, and novel agents being increasingly applied in the treatment of hematological malignancies [7, 8]. As one of the HMAs, DAC has garnered attention, and this study offers a comprehensive evaluation of its impact when used in conditioning regimens for patients with AML and intermediate‐ to high‐risk MDS in remission. Our findings indicate that while DAC conditioning was associated with a reduced relapse rate for myeloid malignancies during remission, it also resulted in increased transplant‐related mortality (TRM). This may help explain why, despite the higher recurrence rate in the HSCT group, the DAC + HSCT group exhibited greater NRM, with the 5‐year NRM rate at 21.3% (95% CI: 6.9–40.9) for the DAC + HSCT group compared to 18.3% (95% CI: 7.9–32.2) for the HSCT group. Although the DAC group showed a lower recurrence rate, its OS events (including both recurrence and non‐recurrence‐related deaths) remained higher, thereby reducing PFS. Consequently, no significant improvements in OS or PFS were observed. Furthermore, this study provided an in‐depth analysis of hematopoietic recovery and immune function restoration post‐DAC conditioning, with a median follow‐up of 5 years—making it the longest follow‐up study to date evaluating the use of DAC as a preconditioning regimen in remission‐phase AML and intermediate‐ to high‐risk patients with MDS.

DAC has a potent inhibitory effect on the bone marrow; however, its direct application in the remission phase of AML and intermediate‐ to high‐risk MDS remains under‐explored. Tang et al.'s study [13] investigated DAC use in patients with AML exhibiting poor molecular genetic prognoses or in intermediate‐ to high‐risk patients with MDS who had not achieved CR prior to transplantation. Their results showed similar neutrophil and platelet engraftment times in both groups, at 12 and 13 days, respectively. This retrospective study from our center directly compared the impact of DAC on long‐term transplant outcomes in AML or intermediate‐ to high‐risk patients with MDS in remission. In our study, the DAC conditioning group had slightly longer neutrophil recovery and platelet engraftment times compared to the control group. With advances in allo‐HSCT, over half of patients with MDS/AML now have the potential for long‐term survival [14]. Our results were also consistent with Li et al.'s study [15], which reported neutrophil engraftment times of 12 days in the DAC group and 11 days in the control group, and platelet engraftment times of 13 days in the DAC group and 11 days in the control group.

Regarding OS, this study included a higher proportion of patients categorized as high‐risk according to the 2016 WHO classification of myeloid malignancies and the IPSS‐R score. However, there is limited data on the direct application of DAC in patients with AML in CR1. This retrospective analysis from our center compared the long‐term transplantation outcomes in AML or intermediate‐ to high‐risk patients with MDS in remission, with and without DAC conditioning. Our findings indicated that DAC incorporation into the conditioning regimen resulted in a reduction in relapse rates for these patients, although blood cell counts and lymphocyte subpopulation function post‐transplantation did not exhibit significant bone marrow suppression.

While DAC conditioning reduced relapse rates post‐transplant, it was also associated with an increased NRM rate and a higher incidence of infections. These results align with those of Zhang et al., who demonstrated the cytotoxic effects of DAC on myeloid malignancies [8]. In vitro studies have shown that combining DAC with BU‐4HC chemotherapy significantly inhibits OCI‐AML3 cell proliferation [13]. Moreover, Qin's research highlighted the synergistic cytotoxicity between DAC and cytarabine, where DAC‐induced hypomethylation enhances cellular sensitivity to cytarabine [16]. The increased TRM observed in our study may be attributed to the more severe bone marrow suppression induced by DAC.

Our data revealed a 5‐year OS of 51.9% (95% CI: 36.1–74.6) in the DAC + HSCT group, compared to 67.0% (95% CI: 53.7–83.5) in the HSCT‐only group. Additionally, the DAC + HSCT group showed a higher NRM rate (21.3% vs. 18.3%). These findings are consistent with those of Li et al., who reported a 2‐year OS of 55.4%, demonstrating the safety and efficacy of this regimen in elderly patients with AML in CR1 [15]. In contrast, Li et al. focused on using HMAs followed by intermediate‐dose cytarabine (ID‐AraC) as pre‐transplant conditioning in HLA‐mismatched (G‐CSF)‐mobilized donor peripheral blood stem cell transplantation. Their results indicated that the DAC‐intensified mBuCy conditioning regimen improved survival in patients with AML, particularly those undergoing haploidentical HSCT, compared to the standard mBuCy regimen [9]. This may be due to DAC's role in enhancing cell sensitivity to cytarabine in the BU‐Cy regimen, as suggested by Qin's study [16]. In a study of a high‐risk subgroup, the 2‐year OS for the DAC‐intensified regimen and HSCT group was 71.3% ± 10.4%, compared to 45.5% ± 10.6%, although the difference was not statistically significant [17]. Cruijsen et al.'s study introduced an RIC regimen combining 10 days of DAC with fludarabine and 2 cGy total body irradiation, reporting a 53% OS and 47% RFS at a median follow‐up of 443 days (range, 46–821 days) [10].

The addition of DAC to the conditioning regimen effectively reduced the incidence of both aGVHD and cGVHD. In our study, the incidence rates of aGVHD and cGVHD were 33.3% and 14.8%, respectively, in the DAC + HSCT group, compared to 50% and 25% in the HSCT group. In contrast, previous studies, such as Cruijsen et al.'s, reported incidence rates of aGVHD and cGVHD at 27% and 60%, respectively [10]. Specifically, for aGVHD, the DAC group exhibited lower rates of both Grade I–II (29.6% vs. 40%) and Grade III–IV (3.7% vs. 10%) aGVHD compared to the control group, which is consistent with findings from Tang et al.'s study. However, while Tang observed a higher incidence of cGVHD in the DAC group (35.6% vs. 21.5%, *p* = 0.03), our study indicated a trend toward reduced cGVHD in the DAC + HSCT group (14.8% vs. 25%).

Further analysis of NK and Treg cells at day 28 post‐transplantation suggested that patients with Grade III–IV aGVHD exhibited relatively higher proportions of these cells compared to those without aGVHD or with Grade I–II aGVHD. This trend was not observed in the control group, implying that DAC may modulate T‐cell subpopulations through its immunomodulatory effects, potentially enhancing NK cell activity and increasing Treg cell proportions. DAC may influence cellular immunity by inducing FOXP3 expression in Treg cells, which could preserve the GVL effect while mitigating GVHD. Previous research by Cany et al. [18] indicated that DAC and Aza reduced proliferation in THP‐1 and KG1a cell lines and suggested that HMAs could enhance the efficacy of allogeneic NK cells derived from CD34+ hematopoietic stem and progenitor cells (HSPC‐NK cells). Moreover, the Foxp3 locus, heavily methylated in CD4+CD25− T cells but not in CD4+CD25+ Tregs, plays a role in suppressing GVHD. Our findings also revealed a correlation between aGVHD incidence and the percentages of CD3−CD56+ NK cells and CD4+CD25+ Treg cells in the DAC + HSCT group. The increased proliferation of these cells in patients with Grade III–IV aGVHD supports the hypothesis that Treg cells act as inhibitors of GVHD, potentially enabling GVHD suppression while maintaining GVL activity [2].

The increased NRM observed with DAC use was linked to elevated infection rates and myelosuppression. Both age at transplantation and the ECOG performance status were found to significantly influence graft outcomes. ROC curve analysis identified an age cutoff of 31.5 years as a potential predictor for graft outcomes, with optimal sensitivity and specificity for survival prediction. However, the biological and clinical rationale behind this threshold requires further investigation in larger cohorts. Although DAC use did impact long‐term survival and PFS, statistical analysis did not reveal significant differences, which could be due to the small sample size in our study.

This study has several limitations. First, the small sample size in our retrospective cohort may have limited the ability to detect variability between the AML‐MRC group and other groups. Second, while CD3−CD56+ NK cells and CD4+CD25+ Treg cells were assessed at day 28 based on prior studies, a more comprehensive analysis of T‐cell subpopulations may provide deeper insights into the underlying mechanisms. Future research is needed to explore the relationship between T‐cell subsets and GVHD severity. Finally, this retrospective study does not fully address the factors influencing therapy selection, underscoring the need for prospective studies to reduce potential biases in treatment choices.

In conclusion, while the addition of DAC to the preconditioning regimen did not significantly improve outcomes overall for patients with AML and intermediate‐to‐high‐risk MDS in remission, it did show beneficial effects in younger patients. Future studies with larger sample sizes are needed to draw more definitive conclusions.

## Author Contributions

S.Y., S.G., and T.L. analyzed the data and drafted the manuscript. W.Z. contributed to the project implementation. X.Y., G.O., and P.Z. designed the study and supervised the process. Data collection, writing, and revisions were collaborative efforts, and all authors approved the final version.

## Ethics Statement

The study was approved by the Institutional Review Board of the First Affiliated Hospital of Ningbo University (2024‐207RS) and was conducted in accordance with the Declaration of Helsinki.

## Consent

The requirement for written informed consent was waived due to the retrospective nature of the study.

## Conflicts of Interest

The authors declare no conflicts of interest.

## Supporting information


Data S1.

## Data Availability

The data are stored at the First Affiliated Hospital of Ningbo University and can be obtained upon reasonable request or by contacting the authors.
