# Supplementary material for: Impact of Decitabine Conditioning on Allo‐HSCT Outcomes in AML and Intermediate‐to‐High‐Risk MDS Patients in Remission
Source: Cancer Med. 2025 Jul 23;14(14):e71081. doi: 10.1002/cam4.71081 (PMC12284727; doi:10.1002/cam4.71081)
Supplement: Supplementary file 1 — Data S1. [file CAM4-14-e71081-s001.doc]

***Supporting Information***

**Impact of Decitabine Conditioning on Allo-HSCT Outcomes in AML and Intermediate-to-High-Risk MDS Patients in Remission**

Shuling Yu^1, 2‡^, Wanchuan Zhuang^3‡^, Shengfa Gao^1, 2^, Tongyu Li^1^, Xiao Yan^1^, Guifang Ouyang^1*^, Ping Zhang^1*^

^1^Department of Hematology, The First Affiliated Hospital of Ningbo University, Ningbo, 315010, China;

^2^Health Science Center, Ningbo university, Ningbo, 315211, China;

^3^Department of Hematology, Lianyungang Second People’s Hospital, Lianyungang, Jiangsu 222000, China.

^‡^Contributed equally.

**Correspondence:**

Dr. Guifang Ouyang

Email: [fyyouyangguifang@nbu.edu.cn](mailto:fyyouyangguifang@nbu.edu.cn)

Department of Hematology, The First Affiliated Hospital of Ningbo University, Ningbo, 315010, China

Dr. Ping Zhang,

Email: fyyzhangping@nbu.edu.cn

Department of Hematology, The First Affiliated Hospital of Ningbo University, Ningbo, 315010, China

**Supplementary figure 1.** ROC curve analysis showing the optimal age cut-off of 31.5 years.


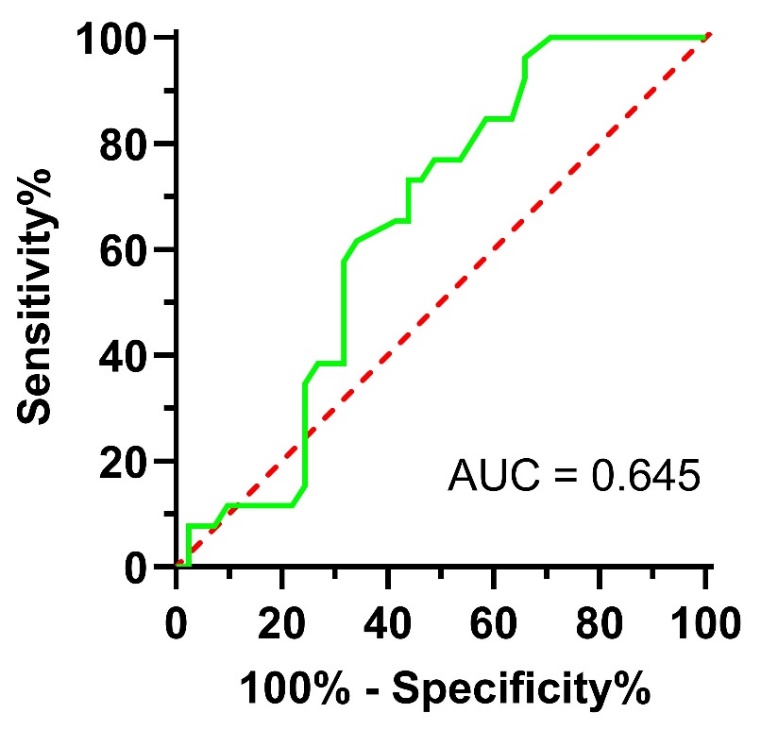


**Supplementary figure 2.** CMV and EBV infection rates across different groups. A-B: Comparison of CMV (A) and EBV (B) infection rates between the DAC-HSCT and HSCT groups.


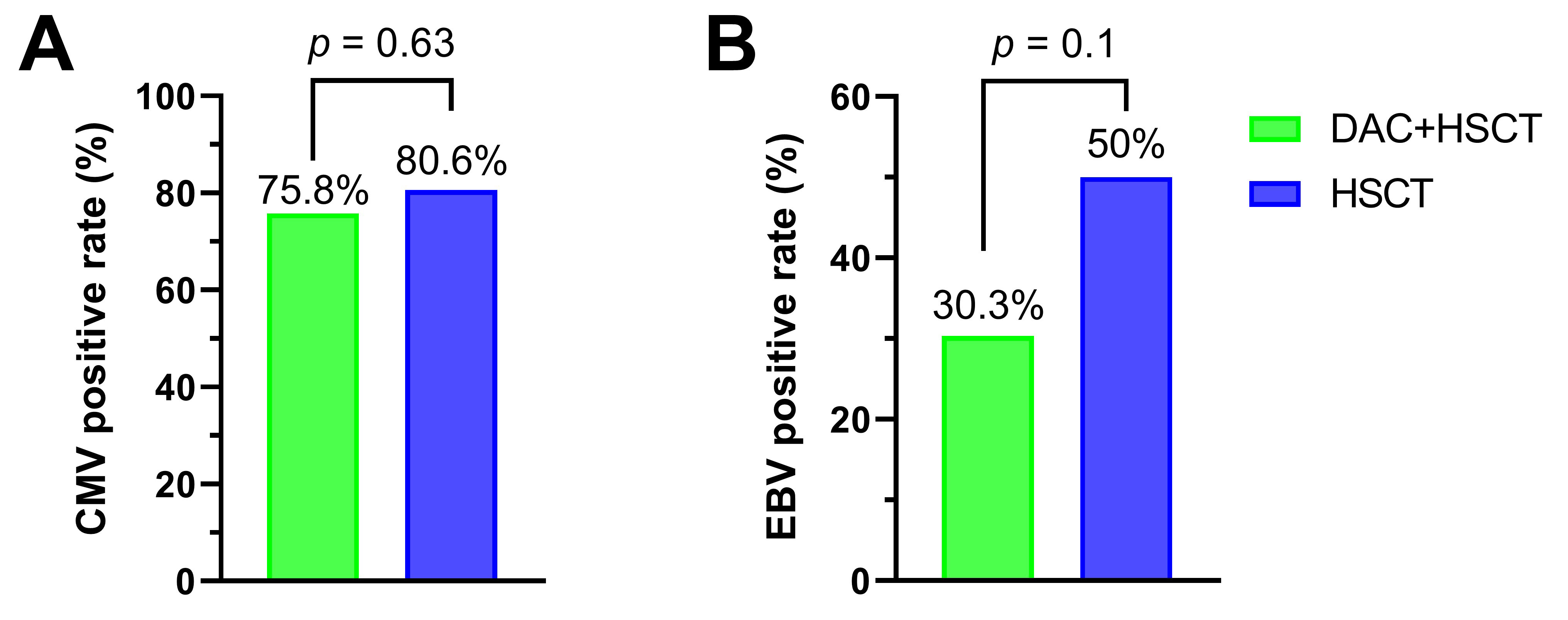


**Supplementary figure 3.** Flow cytometry analysis on Day +28 showed no significant differences between the DAC+HSCT and HSCT groups in terms of CD4+ T cells (16.63% ± 2.23% vs. 16.59% ± 1.25%, p = 0.598), CD8+ T cells (58.67% ± 3.73% vs. 54.88% ± 2.81%, p = 0.422), CD4/CD8 ratio (0.278 ± 0.182 vs. 0.341 ± 0.183, p = 0.148), CD4+CD25+ Treg cells (1.29% ± 0.21% vs. 1.25% ± 0.21%, p = 0.579), and CD3-CD56+ NK cells (13.88% ± 2.36% vs. 14.99% ± 2.15%, p = 0.461).

**
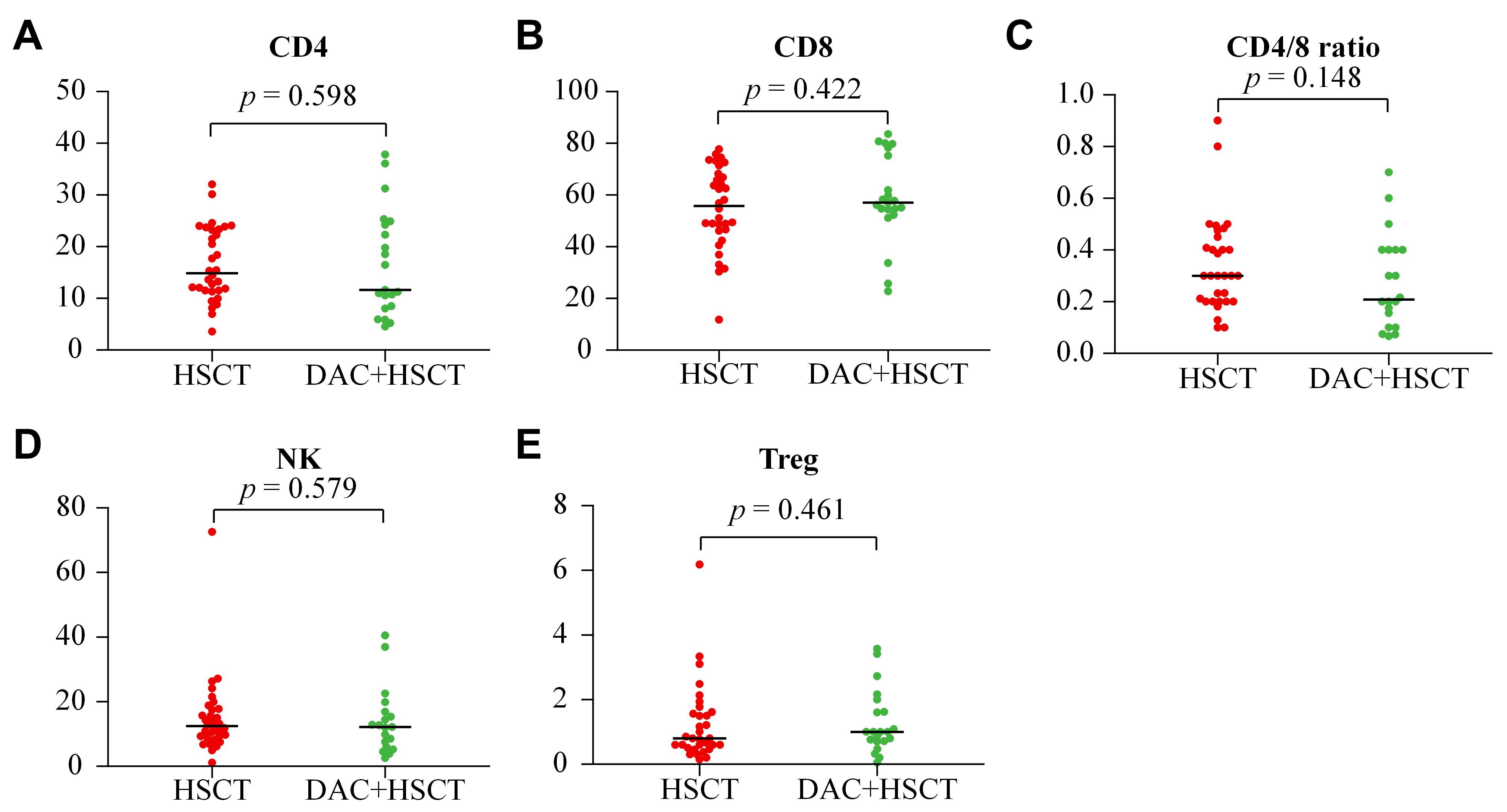
**

**Supplementary figure 4.** Percentages of CD4+CD25+ Treg cells and CD3-CD56+ NK cells in relation to clinical outcomes. (A, C) OS and (B, D) event-free survival for Treg cells and NK cells in the HSCT and DAC+HSCT groups.


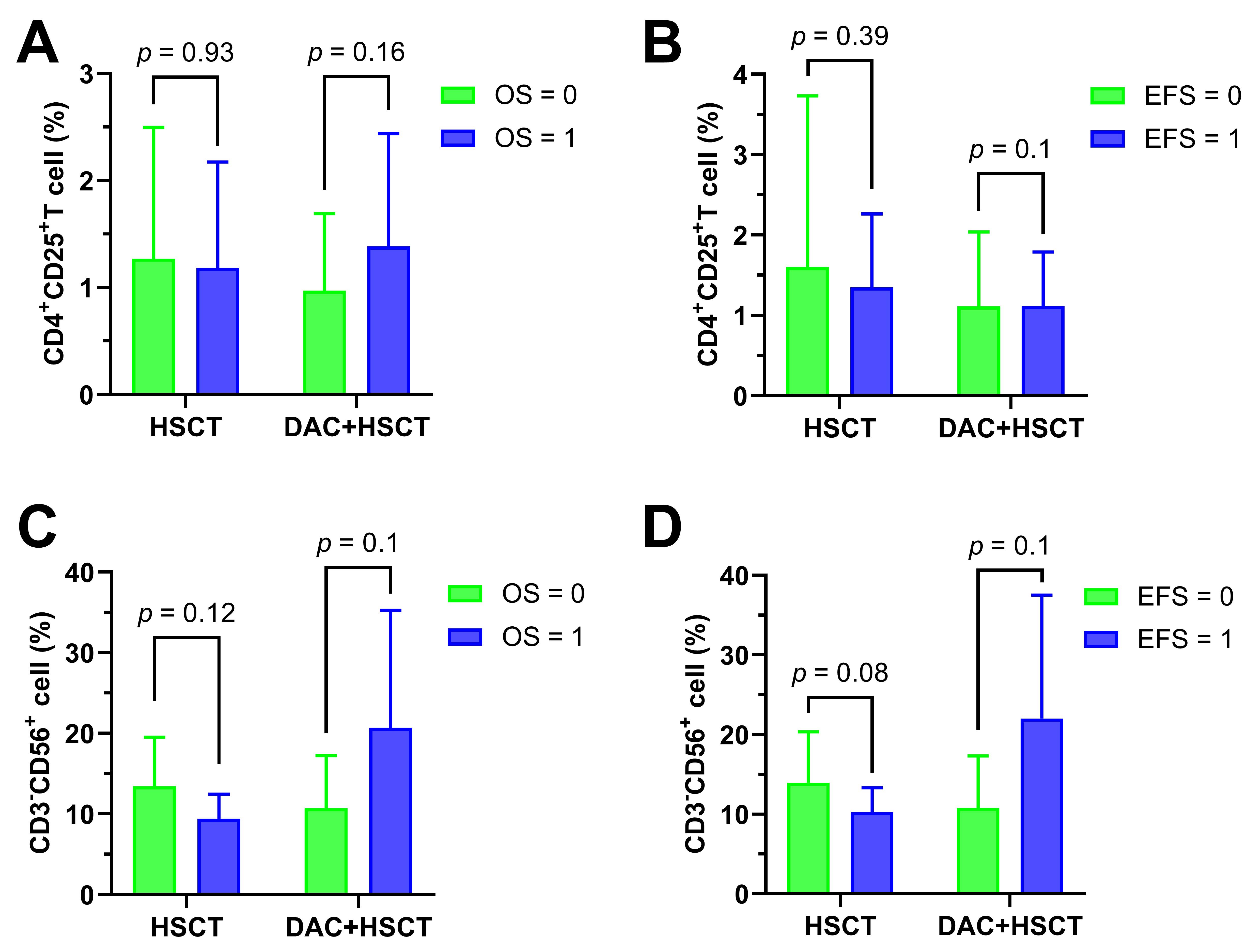


**Supplementary figure 5.** Impact of the DAC-containing conditioning regimen on liver and kidney function, as well as other biochemical markers post-transplantation. We also observed the changes in ALT, AST, gGT, ADA, Cr, and LDH levels from pre-transplantation to day 28 post-transplantation. No significant differences were observed in the trends of these biochemical markers between the two groups (* indicates *p* < 0.05; ns indicates no statistical significance).

**
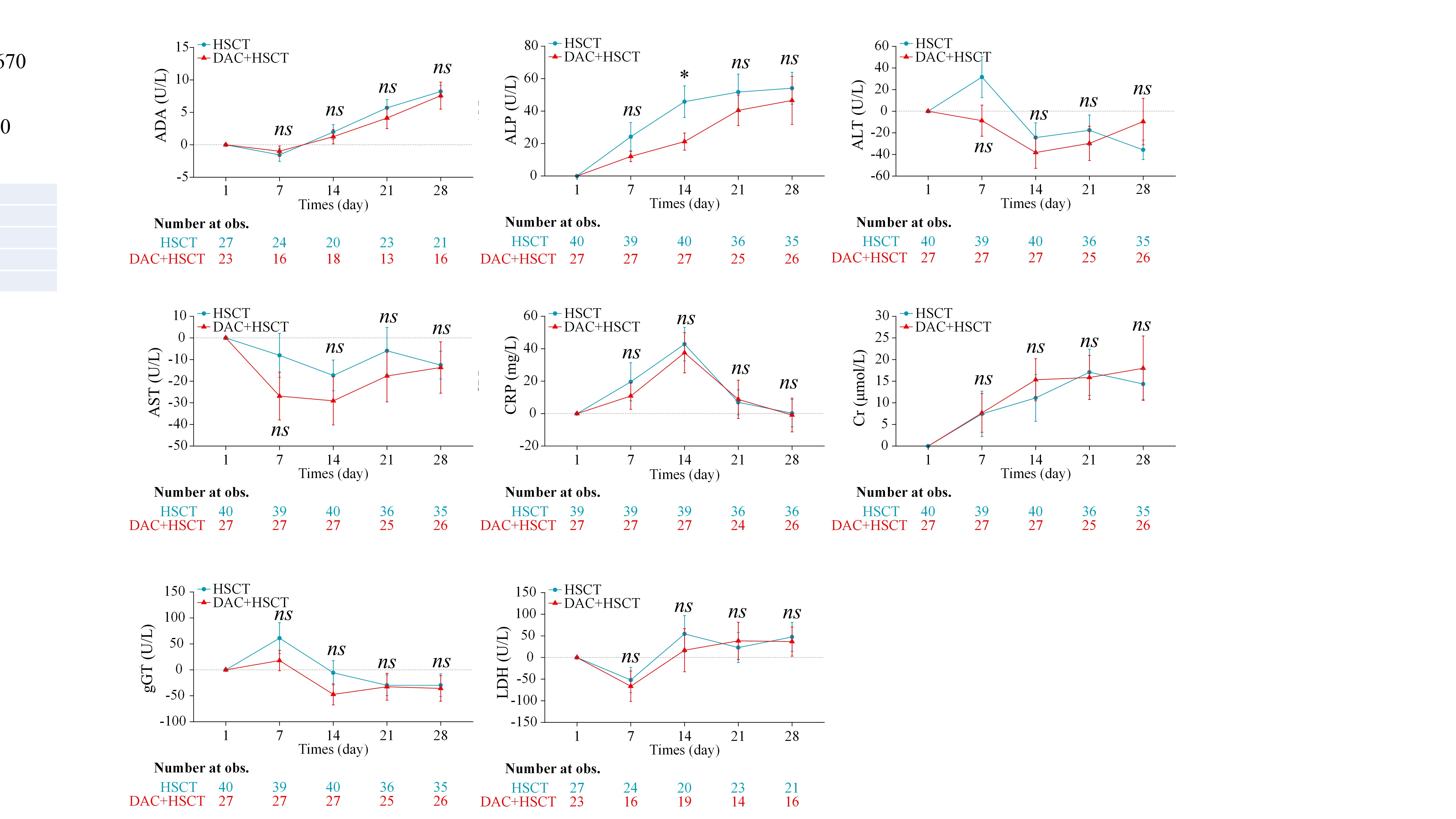
**

**Supplementary figure 6.** Competing risk analysis of RFS, NRM, and GRFS. Considering the impact of competing events, this study performed additional competing risk analysis for RFS, NRM, and GRFS by plotting cumulative incidence curves. The Fine and Gray method was used to compare differences between groups, and competing risk Cox regression analysis was conducted to calculate the risk ratios and 95% CI. The covariates in the models were the same as those in the previously mentioned Cox models. The results indicated no statistically significant differences between the two groups for RFS, NRM, or PFS (HR 0.44 [0.07-2.72], *p* = 0.376; HR 0.48 [0.10-2.36], *p* = 0.367; HR 1.64 [0.71-3.77], *p* = 0.245).


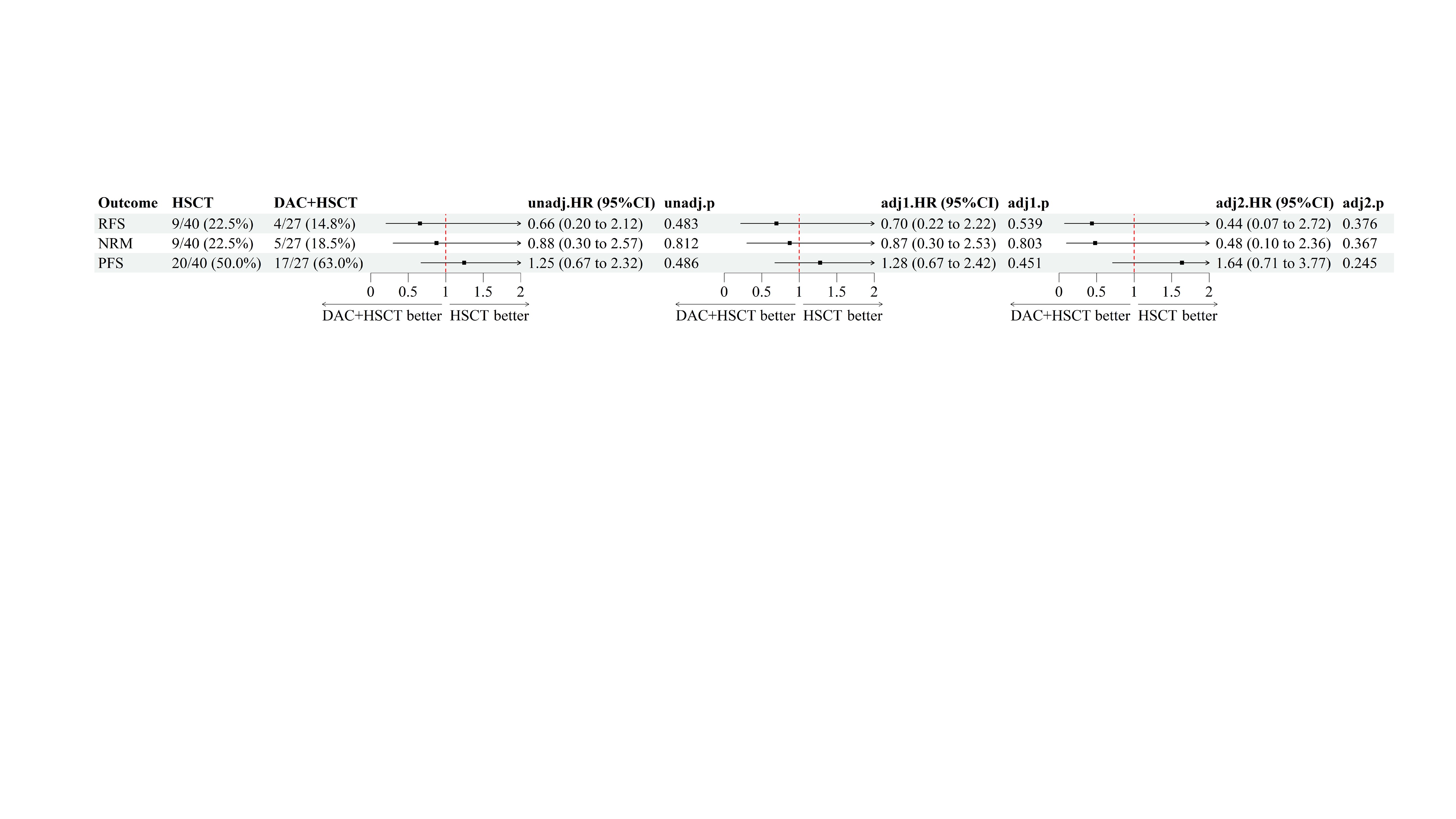


**Supplementary** **table 1.** Incidence and Severity of Acute and Chronic GVHD in DAC+HSCT and HSCT Groups.

| **Characters** | **DAC+HSCT (n = 27)** | | **HSCT (n = 40)** | |  |
| --- | --- | --- | --- | --- | --- |
|  | **n** | **%** | **n** | **%** | ***p*** |
|  |  |  |  |  | 0.363 |
| No aGVHD | 18 | 66.7 | 20 | 50.0 |  |
| I-II aGVHD | 8 | 29.6 | 16 | 40.0 |  |
| II-IV aGVHD | 1 | 3.7 | 4 | 10.0 |  |
| Total aGVHD | 9 | 33.3 | 20 | 50.0 |  |
|  |  |  |  |  | 0.373 |
| No cGVHD | 23 | 85.2 | 30 | 75.0 |  |
| cGVHD | 4 | 14.8 | 10 | 25.0 |  |
